# The Complex Role of Methylation in Regulating Vascular Smooth Muscle Cell Phenotypic States in Vascular Remodeling and Atherosclerosis

**DOI:** 10.3390/biom16060825

**Published:** 2026-06-02

**Authors:** Sanjana C. Basak, Delphine Gomez

**Affiliations:** 1 Pittsburgh Heart, Lung, and Blood Vascular Medicine Institute, University of Pittsburgh, Pittsburgh, PA 15261, USA; sab781@pitt.edu; 2 Department of Medicine, Division of Cardiology, University of Pittsburgh, Pittsburgh, PA 15261, USA

**Keywords:** vascular disease, epigenetics, histone modification, DNA methylation, cell differentiation, cell plasticity, vascular remodeling

## Abstract

Vascular smooth muscle cell (VSMC) control of phenotypic states through regulation of contractile gene expression is critical for vascular homeostasis and for participation in pathological vascular remodeling, such as atherosclerosis. Cohorts of molecular and cellular processes, including transcriptional and post-transcriptional repression of VSMC contractile genes, context-dependent activation of pathological gene sets, proliferation, and migration, coordinately contribute to SMC phenotypic plasticity. Epigenetic (histone post-translational modifications, DNA methylation) and epitranscriptomic (RNA modifications) mechanisms have been implicated in the activation or repression of the VSMC gene repertoire. Among them, methylation exhibits complex, multifaceted, and, in some instances, opposing roles in regulating gene activation. Methylation-mediated epigenetic programming complexity stems from the multiplicity of methylation substrates and enzymes regulating methylation and demethylation. The role and relevance of methylation in regulating VSMC phenotype are often restricted to a given methylation substrate, methylation enzymes, or subsets of genes. The goal of this review is to integrate in vitro and in vivo studies that uncover methylation-mediated VSMC regulation, to assess the overall contribution of methylation-regulating enzymes. We will explore how atherosclerosis-relevant upstream regulatory mechanisms and rate-limiting cofactors of methylation enzymes, including inflammation, metabolism, and hypoxia, affect methylation enzyme activity. Lastly, we will discuss emerging evidence for non-canonical mechanisms by which methylation enzymes may regulate gene expression and their potential role in regulating VSMC phenotype and function.

## 1. Introduction

Vascular smooth muscle cells (VSMCs) are non-striated myocytes that populate the medial layer of most blood vessels, excluding capillaries [1]. VSMCs are essential for regulating blood vessel diameter, blood pressure, blood flow distribution, and vascular integrity through their capacity to contract and dilate, enabled by the lineage-selective expression of a repertoire of contractile cytoskeletal proteins (e.g., smooth muscle alpha-actin, smooth muscle myosin heavy chain), kinases (e.g., Myosin Light-Chain Kinase), and ion channels. In contrast to other myocytes (i.e., cardiomyocytes and skeletal myocytes), VSMCs are inherently plastic cells that can reduce their contractile function and expression of contractile genes, as well as gain proliferative and migratory functions in response to environmental cues. This evolutionarily conserved process, first described as phenotypic switching by Campbell and Campbell [2,3], is an essential property that allows VSMCs to participate in adaptive vascular remodeling, injury repair, and homeostasis. However, sustained pathological cues, such as inflammatory signaling, hypoxia, oxidative stress, and mechanical stress, induce VSMC-dependent maladaptive changes in vascular wall morphology and structural organization (i.e., changes in medial thickness, vessel diameter, and neointima formation) mediated by loss of contractility and enhanced proliferation. Such maladaptive remodeling is found in stent-induced restenosis and is associated with conditions such as hypertension and diabetes [4,5]. Other vascular diseases, such as atherosclerosis, involve more complex alterations in VSMC function and phenotypes. Atherosclerosis is a chronic vascular disease characterized by the formation of a plaque composed of oxidized lipids, inflammatory cells, and resident vascular cells, leading to progressive intraluminal obstruction [6,7,8]. Plaque rupture is the primary driver of myocardial infarction and strokes, which are two of the leading causes of death worldwide. VSMCs participate in plaque formation by extensive oligoclonal proliferation and migration from the media into the neointimal space [9,10]. Classically, VSMCs were thought to form the fibrous cap, protecting against plaque rupture. However, lineage-tracing and single-cell transcriptomic studies in atheroprone mice revealed the extent of VSMC plasticity in atherosclerosis (reviewed in [11]). These studies revealed that most plaque VSMCs lose expression of contractile genes [12,13]. Importantly, they also demonstrated that VSMCs transition to distinct phenotypic states (e.g., fibromyocyte, chondrogenic, foam cell-like, and mesenchymal-like) that can either promote or protect against late-stage atherosclerotic complications [14,15,16]. High-dimensional single-cell transcriptomic sequencing of human atherosclerotic plaques also found that populations expressing SMC-specific genes could be distinguished by fibrotic, osteogenic, and foam-cell-like transcriptomic signatures [15,17,18,19].

Dynamic regulation of the VSMC contractile gene activity is an essential step in physiological and pathological phenotypic plasticity. As extensively reviewed elsewhere [1,20], the VSMC contractile genes, including *ACTA2*, *MYH11*, *TAGLN*, and *CNN1* (encoding smooth muscle a-actin, smooth muscle myosin heavy chain, smooth muscle protein 22 alpha, and calponin 1, respectively) are regulated by a lineage-restricted combinatorial interaction of the transcription factor Serum Response Factor (SRF) and the co-factor Myocardin on CArG (CC[AT]_6_GG) DNA motifs [21,22,23]. The CArG box serves as a binding motif for the ubiquitously expressed transcription factor SRF, which is an activator of growth-response genes like *c-fos*, as well as skeletal and cardiac muscle-specific genes [24]. The cell-specific activation of VSMC marker genes by SRF is mediated by its interaction with the transcriptional cofactor Myocardin, which is exclusively expressed in VSMC and cardiomyocytes [21,25]. Conversely, the transcription factor KLF4 represses VSMC contractile gene expression through binding to G/C elements adjacent to CArG boxes [20,26]. Yet, the CArG-SRF-Myocardin regulatory complex alone is insufficient to explain the lineage-restricted expression of VSMC contractile genes, suggesting the involvement of epigenetic mechanisms. Pioneering studies by Dr. Gary Owens’ group demonstrated that SRF binding to CArG elements in VSMC differentiating from precursors was associated with acetylation of the histone H4 on these regulatory regions, which established histone modifications as a critical regulatory mechanism in VSMC differentiation [27]. Further studies detailed the relationships among histone modification (i.e., acetylation and methylation) occupancy, transcription factor binding, and transcriptional activation of the VSMC contractile gene repertoire [28]. Many epigenetic mechanisms have been implicated in the regulation of VSMC phenotype in vascular remodeling and atherosclerosis pathogenesis [29,30]. Importantly, and as extensively discussed in this work, the complex and specific pathogenesis of atherosclerotic disease is associated with distinct methylation-mediated epigenetic reprogramming. However, the overall contribution of methylation and its regulatory enzymes to VSMC contractile state and plasticity remains elusive due to their multi-layered mechanisms of action, substrate diversity, and the potential impact of atherogenic environmental cues on methylation enzyme catalytic activity.

This review provides an overview of histone, DNA, and RNA methylation involved in regulating VSMC gene expression and phenotypes, with an emphasis on in vitro and in vivo empirical evidence implicating methyltransferases (writers), demethylases (erasers), and methyl-binding protein (readers) in vascular remodeling and atherosclerosis. In that regard, we consider histone, DNA, and RNA methylation enzymes as coordinated components of a dynamic methylation landscape driving VSMC epigenetic programming and pathological reprogramming. Across the VSMC epigenome and epitranscriptome, methylation enzymes exhibit a common dependence on environmental cues implicated in functional vascular remodeling and atherosclerosis, including metabolism, hypoxia, and inflammation. Yet, the putative synergistic, additive, cumulative, or opposing downstream effects of histone, DNA, and RNA methylation suggest an interdependence and hierarchical regulatory framework of these mechanisms. We propose that methylation in VSMC should be examined across the histone/DNA/RNA axis rather than in isolation and discuss the following outstanding questions: (1) What are the methylation writers and erasers regulating VSMC phenotype and function? (2) What are the mechanisms ensuring spatial genomic and transcriptomic selectivity of these enzymes? (3) Which methyltransferases and demethylases regulate VSMC plasticity in atherosclerosis? (4) How do proatherogenic environmental cues influence these enzymes’ activity? While we review evidence directly related to VSMC, we also discuss non-SMC-specific approaches employing methylation enzyme inhibitors (“epidrugs”) in atherosclerosis preclinical studies and their effects on lesion cells.

## 2. Histone Methylation, Methyltransferases and Demethylases

### 2.1. Histone Methylation: Function, Writers, and Erasers

Histone proteins are fundamental constituents of the nucleosome, the repeating chromatin subunit. A nucleosome is formed by 146 base pairs of DNA wrapped around a histone octamer core, comprising an H3–H4 tetramer and two H2A-H2B dimers [31]. Lysine and arginine residues on the N-terminal tail or globular core of histone proteins serve as scaffolds for dynamic post-translational modifications (PTMs) that regulate chromatin structure, DNA accessibility, and gene activity. PTMs are enriched at various genomic loci, often involved in regulating transcriptional activity, such as active genes and enhancers [32]. Histone lysines can be mono-, di-, or tri-methylated; the location of the methylated lysine (histone N-terminal tail or globular core) and level of methylation determines their function in chromatin remodeling and gene activation/repression [33]. Classically, active promoters are marked by H3K4me3, whereas enhancers exhibit H3K4me1 enrichment [34]. H3K27me3 and H3K9me3 are repressive marks that promote heterochromatin formation [32]. The unique network of histone modifications that decorate the PTM landscape at a given time contributes to a highly specific epigenetic state. This “histone code,” as termed by Jenuwein and Allis, contributes to gene activation and silencing and thereby regulates cell function [35]. The histone methylation landscape is spatially and temporally regulated by epigenetic modifiers recruited to the chromatin [32]. Enzymes termed “writers” catalyze the formation of PTM, “readers” facilitate recognition of the PTM, and “erasers” catalyze the removal of the epigenetic mark. The histone lysine methyltransferases include the SET domain-containing proteins and the DOT1-like proteins [33]. The histone lysine demethylases comprise the nuclear amine oxidase family and the jumonji C-domain-containing iron-dependent dioxygenases (JMJD) [36,37]. Histone methylation enzymes exhibit substrate specificity and enzymatic activity that determine the degree of methylation [33].

### 2.2. Regulation of VSMC Contractile Gene Expression and Proliferation by Histone Methyltransferases and Demethylases

**Figure 1:**
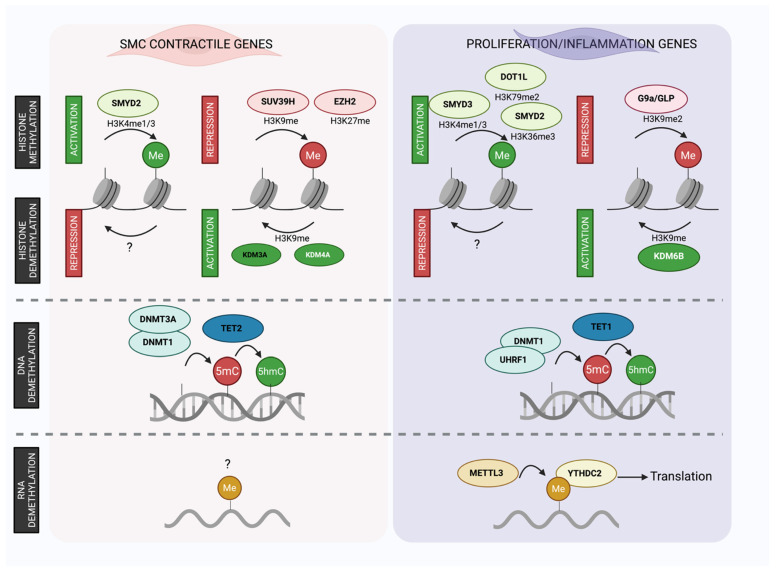
Regulation of VSMC phenotypic transition and plasticity by histone, DNA, and RNA methyltransferases and demethylases. Schematic representation of the regulation of contractile and proliferation genes by histone, DNA, and RNA methylation in the VSMC. Enrichment in activating (H3K4me3) or repressive (H3K9/27me) regulates the activation or repression of SMC contractile genes via SMYD2-, SUV39H1-, and EZH2-dependent methylation. Similarly, DNMTs and TET2 regulate the DNA methylation state on these loci. Genes promoting SMC dedifferentiation, proliferation, and inflammatory state are also activated by HMTs, HDMs, and TET1 during phenotypic transitions. In addition, pro-dedifferentiation and pro-inflammatory transcripts are subjected to METTL3-dependent methylation, which enhances their stability and translation. Question marks indicate that the methylation enzymes responsible offor the given reactions have not been identified.

VSMC contractile gene promoters are decorated with histone methylation consistent with their activation state; while H3K4me3 is present when these genes are activated, its occupancy is markedly reduced during phenotypic modulation-associated repression with subsequent deposition of H3K9 and H3K27 methylation [12,28]. While multiple families of methyltransferases, demethylases, and binding proteins are implicated in mediating the methylation of these histone residues, few have been identified to directly regulate VSMC contractile gene expression, highlighting gaps in our understanding of the upstream mechanisms that dictate the histone methylation landscape in VSMC (Figure 1, Table 1). SMYD2, which promotes an active chromatin conformation through H3K4 and H3K36 methylation, has been shown to promote a contractile and differentiated phenotype by enhancing the enrichment of Myocardin, SRF, and H3K4me1/3 (but not H3K4me2) in the CArG regions of *Acta2* and *Myh11* gene promoters through interaction with the transactivation-domain-containing myocardin C-terminal [38]. Non-inducible VSMC-selective (*Tagln*-Cre) SMYD2 KO was associated with increased neointima size and intima-to-media ratio following wire-induced carotid artery injury. This pro-differentiation effect of SMYD2, however, contrasts with other reports supporting a pro-dedifferentiation and proliferation function [39]. Inducible SMC-specific (*Tagln*-Cre-ER^T2^) SMYD2 overexpression promoted neointima expansion and VSMC proliferation after carotid artery injury via SMYD2-mediated enrichment of H3K36me on *HDAC3*. These contrasting findings regarding SMYD2’s role in VSMC plasticity may be attributable to methodological differences or to opposing regulation of chromatin states by SMYD2. Methodologically, Zhou et al. employed a non-inducible SMYD2 KO mouse model. The pro-differentiation effects on SMYD2 in that context may be due to the enduring consequences of SMYD2 ablation in VSMC during development. Supra-pathophysiological levels of SMYD2 overexpression can also impact enzyme/substrate stoichiometry. A similar dichotomy of SMYD2’s role in cardiomyocytes was shown in zebrafish heart development. Contrasting results found that cardiac-specific SMYD2 KO correlated to atrium and ventricle malformation with tail deformation, while cardiac defects were not observed in a global SMYD2 KO zebrafish model [40,41,42]. Thus, studies are needed to determine the genome-wide and context-dependent occupancy of SMYD2 in VSMCs and how it relates to the H3K4me1/3 and H3K36 methylation landscapes. Furthermore, its potential involvement in VSMC phenotypic modulation toward pro-atherogenic or atheroprotective states has not been evaluated in preclinical atherosclerosis models. Despite the expected key role of H3K4 and H3K36 methylation in positively regulating VSMC contractile gene expression and VSMC differentiation, the role of other major HMTs remains unexplored.

In contrast with activating histone methylation, several central HMTs responsible for the deposition of repressive histone methylation (i.e., H3K9 and H3K27 methylation) have been implicated in contractile gene repression. The methyltransferase EZH2 promotes a repressive chromatin state at VSMC contractile genes by depositing H3K27me3. In a wire-induced carotid artery injury model, oral treatment or perivascular application of the EZH1/2 inhibitor UNC1999 repressed H3K27me3 in neointimal cells and significantly attenuated neointimal formation [54]. In thoracic aortic aneurysm, inhibition of EZH2 by GSK343 prevents aortic dilation and ameliorates ECM organization and VSMC contractile gene expression in *Fbn1^C1039G/+^* (Marfan) mice [49]. Mechanistically, EZH2 recruitment to the VSMC contractile genes is secondary to the formation and binding of a ternary complex consisting of the long non-coding RNA MALAT1, the histone deacetylase HDAC9, and the chromatin remodeling enzyme BRG1 [50]. In atherosclerosis, evidence suggests that retinoic-acid signaling mediates EZH1/2 binding to genes associated with VSMC stem-cell-like state and promotes their repression, potentially inhibiting the further transition of VSMC into fibromyocytes or chondrogenic states [71]. EZH2 has also been shown to promote VSMC proliferation and migration by stabilizing circHECTd [72]. Further studies employing SMC-specific EZH2 KO models may elucidate a more precise mechanism of EZH2 repression of contractile genes and regulation of VSMC plasticity. The methyltransferase SUV39H1 establishes a repressive chromatin conformation through deposition of H3K9 methylation on VSMC contractile genes. SUV39H1 mediates H3K9me3-dependent contractile gene repression through subsequent increased repressive DNA methylation content, reduced chromatin accessibility, and inhibition of the H3K9me3 demethylase KDM4A [46]. Consistent with these studies, SMC-specific and inducible KO of Suv39h1 resulted in a decrease in neointima formation after carotid and femoral ligation-induced vascular injury [47]. These observations suggest that SUV39H1 is a central mediator of vascular injury-induced dedifferentiation and proliferation in vivo, although the lineage tracing studies preclude the definitive assessment of SUV39H1’s impact on VSMC fate in vivo. Another H3K9 demethylase, KDM3A (Jmjd1a), counteracts SUV39H1-induced gene repression by binding CArG-containing regions of the *ACTA2* and *TAGLN* promoters and enhances their activation by interacting with myocardin-related transcription factor A (MRTF-A) [55]. KDM3A knockdown results in increased enrichment of H3K9me2 at VSMC contractile genes.

Together, the epigenetic equilibrium between EZH2/SUV39H1 and KDM3A/4A regulates VSMC phenotypic transition and participation in adaptive vascular remodeling. Yet, the cooperation, redundancy, and interdependence of these enzymes need to be further investigated. Another important understudied aspect of cue-dependent histone methylation enrichment and removal is its temporal and persistent nature. Indeed, several lines of evidence support the existence of epigenetic memory mechanisms in VSMCs. Firstly, our group and others reported that H3K4me2 is stably present on contractile genes in both contractile and dedifferentiated VSMC [12,73,74,75]. Using VSMC fate-mapping mice, our group found that H3K4me2 enrichment on the VSMC contractile genes was retained in ACTA2- VSMC and ACTA2-/LGALS3+ VSMC in atherosclerotic plaques, suggesting this histone methylation signature is stable in subsets of lesional VSMC. These observations suggest that H3K4me2 could serve as a memory mechanism, facilitating VSMC redifferentiation and retention of lineage identity upon removal of certain cues. In vitro and in vivo epigenetic editing, involving the selective demethylation of H3K4me2 on VSMC contractile genes, is associated with a profound loss of lineage identity and contractility [73]. Locus-specific H3K4me2 demethylation also markedly enhanced VSMC plasticity. Yet, the precise mechanisms and the HMT/HDM interplay that regulate the histone methylation landscapes on the VSMC contractile genes remain incompletely elucidated. Similarly, the genome-wide persistence and alteration in the H3K4me2 landscape, and the consequences for VSMC phenotypic states in atherosclerosis, remain to be elucidated. Secondly, seminal studies by Dr. Rama Nataraja’s group have provided compelling evidence for an epigenetically mediated metabolic memory. VSMC extracted from db/db mouse aortas exhibited a persistent increase in pro-inflammatory gene expression, coupled with reduced H3K9me3 occupancy at these loci in the absence of a hyperglycemic milieu [76]. Persistence of altered chromatin accessibility and gene expression was detected in VSMC from diabetic mice treated with dapagliflozin, which ameliorated glucose tolerance [77]. VSMC developmental, physiological, and pathological histone methylation-mediated transcriptional memory should be further investigated as it is likely to profoundly influence VSMC fate in the context of chronic diseases, like atherosclerosis and diabetes, and impact therapy efficacy.

Other HMTs and HDMs have been implicated in the VSMC transition from a contractile, quiescent state to a non-contractile and proliferative phenotype. However, these associated HMTs/HDMs do not directly regulate the VSMC contractile gene histone methylation landscape (Figure 1). The H3K4me1/3 methyltransferase SMYD3 has been shown to be enhanced by PDGF-BB stimulation in rat aortic SMCs [43,44]. SMYD3-silenced VSMCs exhibit a differentiated phenotype characterized by reduced migration, proliferation, and H3K4me3 enrichment on the *PCNA* promoter [43]. In vivo global *Smyd3*^−/−^ mice exhibited a marked reduction in neointimal formation. Perivascular treatment with siSmyd3, associated with balloon-injured carotid arteries in rats, reduced proliferative marker expression and neointima formation. Long et al. also reported that SMYD3 recruitment and H3K4me3 enrichment on the *Parp16* promoter mediated increased PARP16 expression, the Unfolded Protein Response, and ER stress during VSMC phenotypic modulation [44]. Together, this body of work provides evidence that HMTs/HDMs function as components of a complex methylation regulatory network through the regulation of SMC contractile and pro-dedifferentiation genes.

### 2.3. Genetic and Pharmacological Targeting of Histone Methylation Regulatory Enzymes in Atherosclerosis

While the involvement of histone methylation in regulating the methylation status of VSMC contractile genes has been explored in vitro, their roles in VSMC plasticity and atherosclerosis pathogenesis remain to be clarified. Pathological studies in large biobanks of human atherosclerotic plaques reported decreased H3K9 and H3K27 methylation and increased H3K4 methylation in ACTA2-positive plaque areas compared to healthy vessels by histochemistry [78]. Importantly, this approach does not allow for evaluating the abundance of histone modifications in lesional VSMC that have downregulated their contractile repertoire. In VSMC lineage tracing *Apoe*^−/−^ mice, a marked decrease in H3K9me2 was observed in VSMC residing in the medial, core, and fibrous cap areas of atherosclerotic carotid arteries [48]. Pharmacological (UNC0638) and genetic inhibition of the H3K9 dimethyltransferases G9A/GLP enhanced VSMC expression of pro-inflammatory gene networks in vitro and in vivo, demonstrating that G9A/GLP regulates the VSMC pro-inflammatory phenotype. A few in vivo studies have directly examined the functional role of HMTs and HDMs in VSMC during atherosclerosis through cell-specific loss-of-function approaches. SMC-specific and inducible KO of DOT1L (H3K79 methyltransferase) resulted in reduced lesion formation and leukocyte infiltration, as well as augmented plaque stability compared to wild-type control [45]. In vitro studies revealed that DOT1L promotes a VSMC pro-inflammatory phenotype by mediating the enrichment of the activating histone modification H3K79me2 at pro-inflammatory gene promoters, including *Nfkb1*, *Nfkb2*, and *Ccl2*, thereby accounting for decreased immune cell recruitment to the plaque. Yet the impact of DOT1L modulation on VSMC plasticity in atherosclerosis was not addressed.

Despite the paucity of in vivo mechanistic studies examining how HMTs and HDMs regulate VSMC fate in atherosclerotic plaques, many preclinical studies have evaluated the potential therapeutic benefits of epidrugs [79,80]. FDA-approved inhibitors of EZH2 have been extensively used to treat cancer and inflammatory disease [81,82]. In preclinical prevention studies, treatment with the EZH2 inhibitor GS126 markedly reduced plaque development [53]. Interventional treatment with Tazemetostat for 6 weeks was associated with a reduction in plaque burden, lesion size, and macrophage content, while the fibrous cap thickness was increased [83]. However, the impact of EZH2 inhibition on VSMC was not assessed. EZH2 overexpression is associated with increased H3K27me3 level and enhanced accumulation of total cholesterol and triglycerides in foam cells [51,52]. Together, while non-specific EZH2 inhibition seems protective against atherosclerosis development and progression, its impact on VSMC fate and other vascular cell types in atherosclerosis remains unclear. Intervention studies consisting of the administration of the specific LSD1 (H3K4me1/2 and H3K9 demethylase) inhibitor GSK2879552 between 10 and 14 weeks of a high-fat diet in *Apoe*^−/−^ mice showed reduced plaque burden [56]. Together, these studies highlight the therapeutic potential of HDM inhibitors but also the lack of rigorous evaluation of the effects of these drugs on VSMC phenotypic states during atherosclerosis progression.

## 3. DNA Methyltransferases and Demethylases

### 3.1. DNA Methylation: Function and Regulation

DNA methylation refers to a conserved reaction across eukaryotes that involves the covalent addition of a methyl group to the C5 carbon of cytosine (5-methylcytosine or 5mC), although adenine methylation has also been reported [84,85]. Cytosine methylation regulates gene expression and chromatin organization. DNA methylation of promoter and enhancer regions, particularly in CpG islands, is associated with transcriptional repression and enhancer inactivation [86]. DNA methylation can also occur in gene bodies, where it is linked to gene activation and to the suppression of cryptic transcription start sites [87]. DNA methylation is mediated by the DNMT family of DNA methyltransferases, which includes DNMT1, DNMT3A, DNMT3B, and DNMT3L [88]. While DNMT3A and DNMT3B are responsible for de novo DNA methylation, DNMT1 maintains established DNA methylation patterns. DNMTs contain a conserved C-terminal catalytic domain and distinct DNA-binding domains that recognize hemi-methylated CpG sites (DNMT1) or unmethylated H3K4 (DNMT3A/B) [89,90]. The ferrous iron (Fe(II)), a-ketoglutarate-dependent Ten Eleven Translocation (TET) dioxygenase family serves as a DNA demethylase. TET1, TET2, and TET3 catalyze the conversion of 5mC to 5-hydroxymethylcytosine (5hmC), which can be further oxidized to 5-formylcytosine (5fC) and 5-carboxylcytosine (5caC) [91,92]. The unmodified cytosine is ultimately restored through base excision repair mediated by thymine DNA glycosylase (TDG) [90]. 5hmC is enriched at active enhancers and transcribed gene loci [93]. Besides serving as intermediates in active DNA demethylation, 5mC and 5hmC can also function as stable epigenetic marks and be recognized by readers such as UHRF1/2, MeCP2, and the MBD3/NURD complex [93]. These readers further regulate gene expression by recruiting co-activators or co-repressors [93]. Interestingly, DNMTs have been shown to potentially bind and interact with 5hmC in the absence of SAM [94,95].

### 3.2. Regulation of VSMC Contractile Gene Expression and Proliferation by DNMTs and TETs

Compared with studies investigating the HMTs and HDMs that control the histone methylation landscape of the VSMC contractile gene repertoire, the direct involvement of the DMNTs and TETs at these loci is well-characterized (Figure 1). DNA methylation of CpG sites in the promoter–enhancer regions of VSMC contractile genes has been implicated in their repression. Studies by Dr. Christopher Mack’s group first identified that 5mC abundance on the VSMC contractile gene promoters inversely correlates with gene expression and VSMC differentiation status [96]. In this context, DNA methylation regulates RBPJ binding and transcriptional repression of VSMC contractile genes, and is associated with VSMC loss of contractility and dedifferentiation. The VSMC contractile gene transcriptional activity is also regulated indirectly through DNA methylation-mediated control of transcriptional master regulator expression. For example, *PTEN* (phosphatase and tensin homolog), whose gene product potentiates SRF binding on CArG boxes, is dynamically methylated during VSMC phenotypic switching [97]. A drug screen performed on cultured VSMC identified 5-Azacytidine (5-aza-CR), a DNMT inhibitor, as a potent negative regulator of PTEN expression [57]. 5-Azacytidine induces a marked, PTEN-dependent promotion of VSMC contractile phenotype in vitro and prevents neointima formation in vivo [57,58]. The G-protein-coupled receptor 2 (GRK2) has recently been identified as a key regulator of DNMT1 expression and, subsequently, of contractile gene DNA methylation during VSMC phenotypic modulation. The enhanced expression of G protein-coupled receptor kinase 2 (GRK2), observed in dedifferentiated VSMC and atherosclerotic lesions, promotes GRK2-mediated phosphorylation of DNMT1, thereby protecting the DNA methyltransferase from protein degradation [58]. SMC-specific deletion of GRK2 limited vascular injury-induced neointima formation. This phenotype resembled AAV-mediated delivery of shDNMT1 and 5-Azacytidine treatment. Beyond the effect of DNMT1 on PTEN mentioned above, Cleavage Under Targets & Tagmentation (CUT&TAG) profiling of DNMT1 genome-wide occupancy revealed the direct binding of the methyltransferase on the VSMC contractile gene promoters, including *Myh11*, *Cnn1*, and *Acta2*. Interestingly, Focal Adhesion Kinase, a central component of the focal adhesion complex, influences the DNA methylation profile of the VSMC contractile genes in a catalytic activity-independent fashion. Treatment with FAK enzymatic inhibitor VS-4718 correlated to a significant decrease in 5mC content within the promoters of contractile genes *Tagln* and *Myh11* in mouse aortic SMC and potentiation of VSMC contractile state [59]. Mechanistically, non-activated, unphosphorylated FAK localizes within the nucleus, interacts with DNMT3A, and promotes its E3 ligase TRAF6-mediated ubiquitination and degradation [59]. In vivo SMC-specific FAK kinase-dead mice and mice treated with the FAK enzymatic inhibitor VS-4718 exhibited reduced neointimal hyperplasia, DNMT3A expression, and 5mC content upon wire-induced injury compared to the controls [59,98]. A shDNMT3A mouse model also showed reduced neointimal hyperplasia and 5mC levels following wire-induced injury compared to controls [59]. Together, these studies highlighted the role of DNMTs in dynamically regulating the DNA methylation landscape on the VSMC contractile genes, as well as their transcriptomic activity. Of note, both maintenance (DNMT1) and de novo (DNMT3A) DNA methyltransferases have been implicated in this process in vitro and in vivo, although no studies have described the effects of SMC-specific deletion of DNMT3A or DNMT3B on vascular remodeling. Furthermore, DNMTs and TETs are reciprocal components that maintain DNA methylation homeostasis through the addition and oxidation of 5mC, respectively. Despite this counterbalancing methylation/demethylation activity, the VSMC literature examining the role of DNMTs in VSMC plasticity and participation in vascular remodeling lacks appropriate investigation into the opposing methylation dynamics of TETs. Examining DNMT and TET function as a coordinated interaction highlights the complexity of the methylation landscape as a concerted, dynamic system.

Active DNA hydroxymethylation and demethylation by the TET enzymes also play a major role in regulating VSMC contractile gene expression and VSMC phenotypic state. In particular, TET2 has been identified as a central master epigenetic regulator of VSMC differentiation. TET2 is the most abundantly expressed of the TET enzymes in vascular SMC, while its expression level increases with pro-contractility (rapamycin) and decreases with pro-dedifferentiation (PDGF-BB) cues [60]. Seminal studies by Dr. Kathleen Martin’s group demonstrated that TET2 is required for maintenance of the VSMC contractile phenotype [60]. TET2 knockdown inhibited rapamycin-induced contractile gene expression and increased VSMC proliferative capacity by DNA hypermethylation of *MYOCD*, *SRF*, and *MYH11*, and by reduced 5hmC levels at these genomic loci. In contrast, TET2 overexpression promoted hCASMC contractile gene expression and morphology, increased global 5hmC enrichment, and reduced dedifferentiation gene expression. TET2 recruitment to the VSMC gene repertoire not only governs DNA hydroxymethylation but also controls the histone modification patterns on these genes. TET2 knockdown was associated with decreased H3K4me3 occupancy and a concomitant increase in H3K27me3 enrichment. The exact mechanisms by which TET2 influences the histone methylation landscape remain unknown. Histone modifications also influence TET2 recruitment. H3K4me2 facilitates TET2 recruitment to VSMC contractile gene chromatin [73]. H3K4me2 epigenome editing on these loci led to the marked reduction in 5hmC and TET2 recruitment. These studies also demonstrated a genome-wide co-occupancy of H3K4me2 and TET2. Lentivirus-mediated TET2 knockdown in injured femoral arteries significantly increased neointimal area, whereas TET2 overexpression suppressed neointimal hyperplasia compared to the control [60]. Similarly, inducible SMC-specific TET2 deletion was associated with an exacerbation of neointima formation [99,100]. In vascular transplantation-mediated intimal thickening, TET2 plays a critical protective role against VSMC apoptosis and maintenance of medial integrity. Together, these studies highlight the central role of TET2 in regulating VSMC contractile gene expression and VSMC phenotypic state. Of note, the role of TET1 and TET3 in VSMC has not been clearly established. TET1 has been shown to increase 5mC levels in VSMC cultured in a pro-calcification medium and promote VSMC loss of contractile gene expression, although the effects of TET1 modulation on genome-wide 5hmC occupancy and the relevance of these observations in vivo should be further examined [61]. The role of TET3 in VSMC remains largely unexplored. Of note, lineage-tracing studies demonstrate the expression of TET3 in vascular and airway smooth muscle cells [101]. SMC-specific and inducible TET3 KO did not induce obvious morphological abnormalities of the vasculature [101]. In airway SMC, TET3 regulates Polymerase II to limit aberrant transcription initiation within intragenic regions of SMC contractile genes. The relevance of such transcriptional regulation in vascular SMC has not been investigated.

5mC and 5hmC readers also play an important role in regulating the VSMC differentiation state. UHRF1 binds to hemi-methylated cytosine and facilitates the recruitment of DNMT1 for the maintenance of the DNA methylation pattern [90]. UHRF1 expression was significantly upregulated by growth factors, including PDGF-BB [62]. Similarly, an increase in UHRF1+/ACTA2+ cells was observed in atherosclerotic lesions and neointimas compared to healthy vessels. Lentiviral delivery of shUhrf1 reduced neointimal area compared to control carotids. SMC-specific inducible UHRF1 knockout mice exhibited a decreased intimal-to-medial ratio and reduced vascular cell proliferation compared to controls [62]. Mechanistically, UHRF1 directly binds the VSMC contractile genes and mediates their hypermethylation and repression. This loss of contractility was associated with enhanced proliferative capacities.

### 3.3. DNA Methylation and Hydroxymethylation in Atherosclerosis

The genome-wide DNA methylation and hydroxymethylation occupancy in atherosclerotic plaque subpopulations, particularly VSMC, has not been precisely defined. In human atherosclerotic plaques, an increase in 5mC global abundance has been reported [59,102]. Conversely, 5hmC level and TET2 expression were downregulated in human plaques [60]. Similar trends were observed in atherosclerotic plaques of *Apoe*^−/−^ mice [103]. Treatment with 5-azacytidine or its analog 5-aza-2′-deoxycytidine (5-aza-dC or decitabine) was found to be protective against atherosclerosis development and progression [103,104,105]. As a non-cell-specific DNMT inhibitor, 5-azacytine has pleiotropic effects on atherosclerotic plaque morphology and cell composition. It was robustly associated with decreased plaque burden, plaque size, and macrophage infiltration. Interestingly, no difference in ACTA2+ cells within the fibrous cap was observed [103]. Mechanistically, 5-azacytidine dampened macrophage inflammation and blocked shear stress-dependent endothelial cell activation [104,105]. While in vitro studies suggest that DNMT inhibition would favor the maintenance of the VSMC contractile phenotype during atherosclerosis progression [57,103], the impact of 5-azacytidine treatment on VSMC investment and phenotypic states in atherosclerosis has not been evaluated due to the lack of VSMC fate-mapping studies. The need for such studies is supported by a report showing that 5-aza-dC increased human aortic SMC calcification in vitro [106]. Notably, Zhuang et al. attribute the 5-azacytidine mechanism to a “yin–yang” dynamic between DNMT1 and TET2 [103]. DNMT1 enzymatic inactivation by 5-azacytidine may disrupt recruitment of DNMT1 to the TET2 promoter, which could stimulate recovery of TET2 expression and active demethylation. Investigation of a coordinated DNMT1/TET2 mechanism in VSMC may elucidate more precise regulatory mechanisms present in atherosclerosis. The DNA methylation reader UHRF1, which facilitates DNMT1 genomic recruitment, was also significantly elevated in atherosclerotic plaques of *ApoE*^−/−^ mice fed a high-fat diet; increased UHRF1 expression co-localized with ACTA2+ cells and other non-VSMC types [62]. SMC-specific conditional, inducible UHRF1 KO protected against Angiotensin-II-induced abdominal aortic aneurysm, aortic rupture, and atherothrombotic occlusion.

Recent studies on clonal hematopoiesis have associated somatic mutations in *DNMT3A* and *TET2* with increased cardiovascular risk [107]. Functional studies have established that loss of TET2 expression in myeloid cells drives preferential hyperproliferation and exacerbates atherosclerotic progression, with TET2-deficient macrophages exhibiting increased NLRP3 inflammasome activation [108]. The concomitant decrease in TET2 and VSMC contractile gene expression was observed in human atherosclerotic plaques, compounded with the severity of the disease [60]. In the context of transplant-associated vasculopathy and intimal proliferation, TET2 deficiency in VSMC was associated with detrimental enhanced sensitivity to interferon-γ-induced apoptosis and marked medial thinning [100]. Of note, there are no published studies using SMC-specific inducible TET2 knockout in classical murine models of atherosclerosis (i.e., *Apoe*^−/−^, *Ldlr*^−/−^, and PCSK9). Thus, the effect of TET2 loss of activity or loss of expression in VSMC on their phenotypic plasticity and contribution to atherosclerotic plaque progression is unclear. Together, these studies support the central role of DNA methylation in atherosclerosis pathogenesis. Yet, the precise changes in genome-wide methylation and hydroxymethylation occupancy in vascular SMC and their effects on VSMC plasticity and plaque investment have not been precisely and directly investigated.

## 4. N^6^-Methyladenosine (m^6^A) Methyltransferases, Binding Proteins, and Demethylases

### 4.1. RNA Methylation: Function and Regulation

RNA modification is a major epitranscriptomic mechanism that comprises nearly 200 chemical modifications, among which N^6^-methyladenine, 5-methylcytosine, N^1^-methyladenine, and N^7^-methylguanosine are the most well-described. The N^6^-methylated adenine (m^6^A) base is one of the most studied RNA modifications in eukaryotes and has been implicated in VSMC. m^6^A is a chemical modification of mRNA and non-coding RNAs that regulates RNA transcription, splicing, translation, localization, and stability [109,110]. RNA high-throughput sequencing (e.g., MeRIP-seq) revealed that m^6^A is preferentially deposited near stop codons and 3′ UTRs of mRNA transcripts in humans and mice [110,111]. m^6^A levels depend on the organism’s stage of development, cell differentiation state, and response to cellular stress [110]. m^6^A modification occurs co-transcriptionally in the nucleus and, similarly to DNA and histone methylation, is mediated by “writers,” “readers,” and “erasers”. m^6^A is deposited on a RRACH (R = purines; H = A, C, or U) mRNA motif by a writer complex formed by the catalytic subunit methyltransferase-like 3 (METTL3) and the non-catalytic RNA adaptor methyltransferase-like 14 (METTL14) [110,111]. Methyltransferase-like 16 (METTL16) is an additional writer that selectively performs m^6^A methylation on U6 small nuclear RNA (snRNA) and hairpins in 3′ UTR regions, including on MAT2A, which encodes the enzyme methionine adenosyltransferase 2A, synthesizing S-adenosylmethionine (SAM) [111]. Other m6A writers have been implicated in the modification of ribosomal RNA (ZCCHC4) [112]. RNA m6A methylation can be erased by AlkB homolog 5 (ALKBH5) and fat mass and obesity-associated protein (FTO), which demethylate m^6^A and m^6^Am (N^6^,2′-O-dimethyladenosine), respectively [113,114,115]. Of note, TET enzymes have been implicated in RNA 5-hydroxymethylation, adding a layer to their control of gene expression [116]. Several m6A readers have been identified and regulate RNA processing, stability, and translation through m6A-mediated recruitment. Proteins containing YT521-B homology are well-characterized m^6^A readers [111]. Relevant proteins that recognize m^6^A sites in eukaryotes belong to the YTH domain family (YTHDF1-3) and the YTH domain-containing (YTHDC1-2) proteins [111]. YTHDF and YTHDC proteins exert context- and subcellular location-dependent regulation of RNA processing, including transcript degradation (cytoplasmic YTHDF2 [117]), translation activation (cytoplasmic YTHDF1 and YTHDF3 [118,119]), and mRNA splicing (nuclear YTHDC1 [120]).

### 4.2. Role of RNA Methylation in the Regulation of VSMC Contractile Gene Expression and Proliferation

Methyltransferase-like 3 (METTL3), the core catalytic subunit of the N6-methyladenosine (m6A) RNA methyltransferase complex, has emerged as an important epitranscriptomic regulator of VSMC phenotypic state, though its precise role appears highly context- and target-dependent. Increased m^6^A enrichment and METTL3 mRNA and protein expression were detected in VSMCs stimulated with PDGF-BB [66,67]. METTL3 silencing in vitro impaired VSMC synthetic phenotype, proliferation, and migration, and was accompanied by an increase in VSMC contractile gene expression [66]. Several mechanisms downstream of METTL3 have been identified, including the stabilization of *Pi3k* and *Tnfsf10* transcripts, both of which are implicated in VSMC proliferation and migration [66,68]. METTL3 was also shown to methylate the *Pfn1* transcript (encoding Profilin-1), which activates STAT3 signaling and subsequently induces the VSMC proliferative and migratory state [67]. In vivo, METTL3 expression was significantly increased in neointimal arteries of rodent models of neointimal hyperplasia [66,67]. AAV-mediated shMettl3 arteries reduced neointima formation while increasing VSMC contractile marker expression after balloon-induced carotid artery injury in rats [66]. In mice, SMC-specific inducible METTL3 KO similarly reduced neointima formation following vascular injury and arteriovenous fistula formation [67,121]. In apparent contrast, METTL3 was found to be downregulated in stent-implanted coronary arteries during restenosis [122]. This study reported that knockdown of METTL3 in human aortic SMCs facilitated, while overexpression suppressed, VSMC proliferation, migration, and transition to a synthetic phenotype. The lack of consensus could be due to the diversity and context-dependent METTL3-interacting partners, as well as the source of VSMC used in the experiments, possibly suggesting species- or vascular bed-specific effects. Moreover, these two studies differ strikingly in their characterization of METTL3 expression trajectory. While METTL3 was upregulated in response to carotid artery injury, it was downregulated in stented coronary arteries. One possibility is that METTL3 is transiently upregulated in the early acute injury phase (as captured in balloon injury models at 14 days post-surgery) but becomes progressively downregulated as the proliferative phase matures (as might be captured at the stage of established stent-induced neointima). The possibility of a complex temporal and developmental regulation of METTL3 expression is supported by a report showing a transient increase in m^6^A levels in rat aortic SMC treated with PDGF-BB, while METTL3 expression declined at 48 h of treatment [123]. METTL14 also promotes VSMC phenotypic switching and proliferation in vitro and in vivo [63,64]. A proposed mechanism is the methylation-dependent stabilization of the pro-dedifferentiation transcripts Klf4 and Tead1 by METTL14.

The YTHDF m^6^A readers emerge as potent regulators of VSMC phenotypic state. While our understanding of their contribution to VSMC contractility, phenotypic switching, and proliferation is limited, recent studies have established a link between variation in YTHDF expression and these processes. YTHDF1 expression was shown to be significantly reduced during PDGF-BB treatment and after vascular injury [123]. Non-cell-specific lentiviral delivery of YTHDF1 to injured rat carotid arteries notably decreased injury-caused neointima formation and restored ACTA2 and CNN1 expression in the vessel wall’s media [123]. While these studies establish a causal relationship between YTHDF1 overexpression and the promotion of the VSMC contractile state, the impact of SMC-specific YTHDF1 loss-of-function and the mechanisms by which YTHDF1 limits VSMC phenotypic switching have not been established. In VSMC exposed to high glucose, YTHDC2-mediated m^6^A modification stabilized the circular RNA circYTHDC2. circYTHDC2 negatively regulated TET2 expression by targeting the unstable motif in the TET2 3′UTR, thereby promoting the proliferation and migration of VSMCs. This reveals an unexpected epitranscriptomic–epigenetic crosstalk loop whereby YTHDC2-driven m6A modification of a circRNA suppresses TET2, which itself is a master regulator of the contractile phenotype.

### 4.3. Epitranscriptomic Regulation of VSMC Phenotype in Atherosclerosis

Human coronary artery VSMC (HCASMC) cultured with ox-LDL exhibited upregulation of METTL3 [124]. Consistent with these observations, increased METTL3 expression correlates with atherosclerotic plaque severity in human specimens. SMC-specific inducible METTL3 KO markedly reduced atherosclerotic plaque formation in PCSK9-injected, Western diet-fed mice [125]. The diminution in plaque area was accompanied by decreased necrotic core area and lipid accumulation. Remarkably, lineage tracing revealed that METTL3 KO completely prevented VSMC investment into the lesion. In LPS-stimulated macrophages, METTL3 deficiency was associated with enhanced pro-inflammatory response, including upregulation of TNF-a, IL-6, and INOS expression, and increased NO production, suggesting METTL3 could display an atheroprotective role in macrophages [126]. However, METTL3 knockdown was also shown to inhibit IRF-1-induced macrophage pyroptotic death, which suggests that METTL3 may be critical in promoting macrophage cell death in late-stage atherosclerotic lesions, formation of the necrotic core, and plaque progression [69]. Importantly, in vivo, mitigation of atherosclerotic plaque progression was also reported in myeloid cell METTL3 KO mice, with downregulation of pro-inflammatory gene expression [70]. These studies suggest that METTL3 could be an interesting therapeutic target in atherosclerosis, although the overall impact of METTL3 inhibition on atherosclerotic plaque initiation, progression, and stability remains to be evaluated. Importantly, the striking lack of VSMC plaque investment raises questions about the impact of METTL3 deficiency on fibrous cap formation and overall plaque stability. Although non-VSMC can participate in the formation of the fibrous cap (e.g., endothelial cells), the lack of VSMC participation in the fibrous cap is likely to be detrimental and facilitate plaque rupture.

Overall, the contribution of m6A writers, erasers, and readers (beyond METTL3) to atherosclerosis pathogenesis and regulation of VSMC phenotypic state should be further investigated. Indeed, studies on non-VSMCs suggest the implication of other m6A modulators in atherosclerosis pathogenesis. YTHDF2 cooperation with METTL3 is associated with increased cellular and mitochondrial ROS accumulation in inflammatory monocytes, in addition to enhanced pro-inflammatory cytokine release [127]. METTL14 has also been implicated in regulating macrophage inflammation through promotion of foam cell formation and negative regulation of the anti-inflammatory M2 macrophage phenotype [128]. METTL14 has been further shown to promote endothelial cell inflammation and facilitate atherosclerosis pathogenesis through FOXO1 m^6^A modification [65]. Involvement of YTHDF2 and METTL14 in promoting athero-adjacent macrophage and endothelial cell phenotype through inflammatory regulation indicates that epitranscriptomic methylation enzymes beyond METTL3 are likely implicated in VSMC participation in atherosclerosis pathogenesis.

## 5. Modulation of Methyltransferase and Demethylase Expression and Activity by Atherosclerosis-Associated Environmental Cues

Atherosclerosis is a chronic disease characterized by the formation of a complex plaque in which a subset of medial VSMC proliferate and transition to distinct phenotypic states, including fibromyocytes, osteochondromyocytes, and VSMC-derived foam cells [11,14]. VSMC plasticity and atherosclerotic plaque progression are driven by sustained modifications of the vessel’s environment, including inflammation and hypoxia. These environmental cues have well-established consequences for cellular metabolism, which in turn regulate key processes, including proliferation, survival, and phenotypic states. Importantly, histone, DNA, and RNA methyltransferases and demethylases are sensitive to inflammation, hypoxia, and metabolic shifts through variation in their expression or modulation of their catalytic activity. This section will provide an overview of the relationships among metabolism, inflammation, and hypoxia, and the regulation of methylation, as well as the evidence for their implications, or lack thereof, in atherosclerosis and associated VSMC phenotypic modulation. We additionally examine the role of epigenetic and metabolic memory in atherosclerosis and the implication of methylation enzymes as pathogenic signatures.

### 5.1. The Atherosclerotic Plaque Microenvironment

The atherosclerotic plaque microenvironment is modulated by factors such as hemodynamic force, lipid deposition, inflammation, and hypoxia, which induce pathologic changes in vascular cell function. Altered glucose and lipid metabolism initiates endothelial cell, macrophage, and VSMC dysfunction in atherosclerosis [129]. The pro-atherogenic microenvironments perpetuated by the secretion of inflammatory cytokines, growth factors, and lipid mediators stimulate VSMC phenotypic switching into a proliferative and migratory state, and prime their transition into athero-promoting foam cell-like and osteochondrogenic phenotypes [130,131]. Hypoxia and HIF signaling are additional regulatory mechanism that promotes both metabolic dysfunction and chronic inflammation in the atherosclerotic plaque microenvironment. Hypoxia-induced angiogenesis from the vasa vasorum supplies oxygen to the plaque [132]. Nascent vessels also destabilize the plaque and facilitate immune cell infiltration [133]. HIF-1a has been demonstrated to promote lipid accumulation and atherosclerosis pathogenesis by regulating lipid metabolism-associated genes in macrophages [134]. Together, cell metabolism, inflammation, and hypoxia are critical factors regulating the atherosclerotic plaque microenvironment and VSMC participation in atherosclerosis pathogenesis. These environmental factors also modulate the function and expression of epigenomic and epitranscriptomic methylation enzymes, establishing a mechanistic link between the plaque microenvironment and epigenetic regulation of VSMC phenotypic transition and plasticity.

### 5.2. Cell Metabolic State

Histone, DNA, and RNA methyltransferase activity relies on the metabolite SAM as a methyl group donor to facilitate chromatin modification [135]. SAM is synthesized through ATP-dependent methionine condensation by the rate-limiting enzyme methionine adenosyltransferase (MAT) [136,137]. The SAM biosynthesis product S-adenosylhomocysteine (SAH) is hydrolyzed to homocysteine through the one-carbon metabolism pathway, and subsequently converted back to methionine by the methyl group donor L-methyltetrahydrofolate (5-MTHF) [138]. Conversion of homocysteine to methionine maintains a homeostatic ratio between SAM and SAH; SAM/SAH homeostasis is an indicator of cellular metabolic state and directly regulates methyltransferase activity [138]. SAH directly inhibits methyltransferase activity by acting as a competitive inhibitor of SAM [139]. Methionine restriction in six human cell lines was shown to deplete SAM and SAH levels and significantly reduce H3K4me3 enrichment [140]. In addition to MAT reactions, threonine catabolism to glycine and acetyl-CoA, catalyzed by threonine dehydrogenase (TDH), serves as a source for SAM biosynthesis via one-carbon metabolism [141]. Threonine or TDH depletion was found to significantly reduce the SAM/SAH ratio and H3K4me2/3 abundance in mouse embryonic stem cells [141].

JMJD histone demethylases, TET DNA demethylases, and RNA demethylases ALKBH5 and FTO are iron(II)-dependent dioxygenases that require O_2_ as a substrate and α-ketoglutarate (aKG, also known as 2-oxoglutarate) as a cofactor. aKG is an intermediary metabolite of the tricarboxylic acid (TCA) cycle. The initial step of the TCA cycle involves the generation of citrate from acetyl-CoA and oxaloacetate. Citrate is converted into isocitrate and subsequently converted to aKG. Additional steps in the TCA cycle involve aKG conversion to succinyl-CoA, succinyl-CoA conversion to succinate, and the oxidation of succinate to fumarate. Finally, fumarate is converted to malate, and malate is oxidized into one of the initial substrates, oxaloacetate [142]. Two CO_2_ molecules, two NADH molecules, and one FADH_2_ molecule are generated during the TCA cycle. There are several examples of upstream, intrinsic regulation of aKG flux in the TCA cycle. The redistribution of TCA metabolites for cytoplasmic biosynthesis seems to be a positive regulator of aKG flux. Citrate export to the cytosol for biosynthetic purposes activates the anaplerotic process of glutaminolysis, where glutamine is converted to glutamate, and is subsequently oxidized to aKG [142]. In contrast, aKG flux can be diminished by glutamine-dependent reductive carboxylation, where aKG is reduced to glutamate and facilitates citrate generation [143]. Succinyl-CoA can also reduce aKG flux by inhibiting citrate synthase and aKG dehydrogenase [142]. Importantly, aKG-derived metabolites are the most potent inhibitors of histone, DNA, and RNA iron(II), aKG-dependent dioxygenase demethylases. The activity of these enzymes is dependent on the intracellular proportion of aKG to succinate, fumarate, and 2-Hydroxyglutarate (2-HG). In cancer, succinate dehydrogenase deficiency correlated to an increased 5mC/5hmC ratio and enhanced histone methylation, which was reversed by aKG supplementation to culture medium [144]. aKG conversion to 2-HG, and its isoforms L-2-HG and D-2-HG, is independent of the TCA cycle. 2-HG is a competitive inhibitor of aKG and has been shown to inhibit H3K4, H3K9, H3K27, and H3K79 demethylase activity and TET enzyme-mediated DNA demethylation [145].

S-adenosylmethionine (SAM), α-ketoglutarate (αKG), and succinyl-CoA have emerged as important regulators of VSMC functions. While a high-methionine diet has been shown to promote atherosclerotic plaque initiation and development in atheroprone mice [146,147], SAM has been shown to prevent VSMC dedifferentiation by upregulating contractile marker proteins (α-SMA and SM22α) and downregulating synthetic marker proteins (OPN, MMP2, and MMP9) [148]. Additionally, hyperhomocysteinemia, resulting from SAM conversion to SAH and defective methionine regeneration, promotes VSMC proliferation and phenotypic switching [149]. The glutamine–αKG metabolic axis is a critical driver of VSMC proliferation, migration, collagen synthesis, and survival under glutamine deprivation, establishing αKG as the primary mediator of glutamine-dependent VSMC phenotypic modulation [150]. Importantly, αKG treatment in rats and mice remarkably inhibited vascular calcification in a TET2-dependent manner [151]. Taken together, these studies highlight the reliance of VSMC function and epigenetic control on SAM and aKG availability. Altered SAM and aKG metabolism contribute to a broader VSMC metabolic shift that pushes the cell toward the maintenance of a pathogenic environment by promoting VSMC dedifferentiation and impairing methylation enzyme function. The precise contribution of SAM and aKG in mediating VSMC plasticity in atherosclerosis, as well as the precise impact on the multiple and diverse epigenetic mechanisms relying on these metabolites, needs to be further investigated to establish a mechanistic link between metabolic dysregulation and disrupted epigenetic control.

### 5.3. Inflammation

The role of inflammatory pathway signaling on methylation enzyme expression, recruitment, and activity has been established in multiple pathologies. The association between inflammation and methyltransferase/demethylase function is best characterized in macrophages in the context of inflammatory diseases, as well as cancers. Mice infected with *H. felis* were shown to exhibit aberrant DNA methylation concomitant with increased expression of pro-inflammatory genes and DNMT1, while TET1 and TET3 were repressed [152]. TET enzymes have been associated with regulating the inflammatory response of myeloid cells. TET2 plays a central role in inflammation resolution and downregulation of pro-inflammatory genes, including *Il6* [153]. TET3 also displays an anti-inflammatory role and has been shown to inhibit the expression of *Ifnb1* in macrophages [154]. Interestingly, and as discussed in Section 7, TET2- and TET3-mediated repression of pro-inflammatory genes is independent of their DNA demethylase function but relies on non-canonical scaffolding properties leading to the recruitment of Histone Deacetylases (HDACs) on these loci.

Growing evidence links inflammatory signaling to altered DNA and histone methylation in VSMC. Across studies, erosion of repressive H3K9 methylation under inflammatory conditions has been observed [48,76]. H3K9me2 was significantly reduced within atherosclerotic lesions. Harman et al. demonstrated the functional significance of this loss, showing that loss of H3K9me2, mediated by inhibition of the G9A/GLP dimethyltransferase, exacerbates inflammation-induced upregulation of disease-associated genes in vitro and in vivo, partly by facilitating binding of NF-κB and AP-1 transcription factors at specific inflammation-responsive loci [48]. KDM4B-demethylation of RUNX2 has been shown to induce IL-6/STAT3-dependent differentiation of VSMC into osteoblast-like cells [155]. Elucidating the role of inflammatory signaling in modulating histone, DNA, and RNA methyltransferase/demethylase function in VSMC would enhance our understanding of the contribution of epigenetic regulation to atherosclerosis progression.

### 5.4. Hypoxia

Hypoxia influences methylation enzyme expression and activity through direct transcriptional regulation and/or extrinsic regulation of metabolic pathways. Hypoxia modulates the TCA cycle and associated aKG flux through HIF-1α -induced transcription of pyruvate dehydrogenase kinase 1 (PDK1) and lactate dehydrogenase A (LDHA) [156]. PDK1 phosphorylates and inactivates pyruvate dehydrogenase (PDH), which prevents the conversion of pyruvate to acetyl-CoA and reduces TCA cycle flux [157]. Meanwhile, LDHA facilitates the conversion of aKG to L-2-HG under hypoxic conditions [158]. Hypoxia, or reduced oxygen availability, has had demonstrable effects on methylation enzyme function in multiple cell types. Most obviously, demethylases such as JMJD and TET proteins are iron(II)- and a-KG-dependent dioxygenases that require O_2_ to catalyze oxidation reactions. However, there is also evidence that hypoxia induces changes in these enzymes’ expression. KDM3A (JMJD1A) and KDM4B (JMJD2B) have been shown to be significantly upregulated under hypoxic conditions [159,160,161]. In this context, HIF-1α was shown to bind HRE sites in the *KDM3A* and *KDM4B* promoters and promote these genes’ expression. Interestingly, H3K9me2 and H3K9me3, which are targets of KDM3A- and KDM4B-mediated demethylation, significantly increased in abundance with deferoxamine (hypoxia chemical inducer) treatment, despite the noted increase in expression, suggesting an uncoupling of expression and activity. TET1 and TET2 have been shown to have an oxygen K_M_ of approximately 30 M, which would suggest that the TET proteins should remain partially active under low-oxygen conditions [162]. However, 45% and 52% reduction in TET1 and TET2 activity, respectively, have been reported in cancer cell lines under hypoxic conditions [162,163]. While TET expression widely varies as a function of hypoxia in different cell types, HIF-1α can directly upregulate TET2 expression through direct binding to its promoter [164]. These studies illustrate the complex and context-dependent role of hypoxia in regulating methylation enzyme function and highlight possible discrepancies between expression and catalytic activity.

Atherosclerotic plaque expansion contributes to localized hypoxic stress due to increased intercapillary distance, enhanced oxygen consumption, and intraplaque neovascular leakiness [132,165]. Atherosclerotic plaques are robustly hypoxic, especially within the necrotic core, and HIF-1α downstream signaling has been implicated in plaque development [166,167]. Myeloid-specific deletion of HIF-1α protected against atherosclerotic plaque progression through reduction in the necrotic core expansion and macrophage necroptosis [168]. Similarly, VSMC-selective HIF-1α KO prevented plaque formation and lipid accumulation in an accelerated atherosclerosis model induced by transverse aortic constriction (TAC) [169]. Yet the mechanistic relationship between hypoxia/HIF-1α, methylation, and VSMC contribution to atherosclerosis pathogenesis has not been established. Cumulatively, the literature supports an emerging causal link between inflammatory signaling and methylation in pathologies characterized by chronic inflammation.

### 5.5. Epigenetic Control of Inflammatory and Metabolic Memory

Inflammatory and metabolic memory describe mechanisms of functional reprogramming where environmental exposure to pathogenic stimuli alters the cell’s functional state despite eventual reversion to a basal environmental condition. Inflammatory memory can be established when cells encounter an inflammatory stimulus that induces sustained changes in transcriptional response to subsequent stimuli. Metabolic memory develops when cells in an aberrant metabolic environment retain associated functional reprogramming after conditions normalize. These profound and sustained alterations of cell phenotype and function may contribute to reduced efficacy of therapies or risk factor interventions in the treatment of atherosclerosis. A pivotal study by Dr. Rama Natarajan’s group on VSMCs extracted from db/db mice found that H3K9me3 levels were significantly and sustainably decreased at the promoters of key inflammatory genes—including Il6, Ccl2, and Csf1—and that the inflammatory dysregulation underpinned a persistent atherogenic and proinflammatory phenotype even after removal of the original hyperglycemic stimulus [76]. db/db VSMCs also displayed reduced expression of the H3K9 methyltransferase Suv39h1, which may be one of the mechanisms driving the decrease in H3K9me3. Decreased H3K9me3 enrichment at inflammation-associated gene promoters and an associated reduction in Suv39H1 expression establish a cellular epigenetic memory that supports the potential maintenance of a VSMC inflammatory memory. In addition to sustained aberrant H3K9 methylation occupancy, metabolic memory associated with hyperglycemia exposure was observed in diabetic patients and characterized by a sustained differential CpG DNA methylation pattern [170]. Recently, Tanwar et al. used single-cell multimodal profiling to evaluate the transcriptomic and epigenetic state of VSMCs extracted from db/db mice, some of which were treated with the anti-diabetic drug dapagliflozin [77]. VSMCs clustered into 9 subgroups based on SMC contractile phenotype, fibrosis, and inflammation-associated pathways. SMC contractile phenotype-associated pathways were downregulated in VSMC from db/db mice, and were found to remain decreased when db/db mice were treated with dapagliflozin for glycemic control. These findings indicate that metabolic memory is preserved in VSMC despite pharmaceutical intervention and management of disease. Cellular inflammatory memory was detected in patients with severe coronary atherosclerosis, where isolated monocytes exhibited a trained immunity phenotype, which included elevated cytokine production ex vivo [171]. Mouse models that received a Western diet, BCG vaccination, or β-glucan administration demonstrated both trained immunity in circulating monocytes and persistent inflammatory reprogramming of myeloid progenitor cells in the bone marrow [172]. Elucidating the alterations in chromatin conformation that may underpin this persistence in metabolic and inflammatory memory would provide valuable insight into epigenetic mechanisms that prevent successful disease treatment.

## 6. Crosstalk Between Histone, DNA, and RNA Methylation

The establishment of the epigenetic landscape relies on the dynamic coordination of epigenetic and epitranscriptomic mechanisms. The expansion of the “histone code” concept to the interplay among histone, DNA, and RNA “writers,” “readers,” and “erasers” in shaping the epigenetic landscape can be exemplified in VSMC. A pioneering study by Dr. Kathleen Martin’s group demonstrated the central role of TET2 in regulating DNA methylation and hydroxymethylation levels in VSMC contractile genes [60]. Concomitant with DNA methylation, TET2 recruitment at these loci also influenced the abundance of H3K4me3 and H3K27me3, providing evidence that TET2 coordinates its DNA methylation and histone modification landscape. Whether TET2 or modified DNA mediates the recruitment of HMTs or HDMs remains to be determined. Importantly, TET2 recruitment on the VSMC contractile gene itself is dependent on histone methylation. Our group found that TET2 recruitment to the VSMC contractile genes requires H3K4me2 [73]. Gene-selective epigenetic editing of H3K4me2 at the VSMC contractile gene promoters resulted in impaired TET2 binding and DNA hypermethylation. Co-immunoprecipitation studies further established that TET2 preferentially interacts with H3K4me2 compared with other H3K4 modifications. These data provide evidence that H3K4me2 enables dynamic TET2 recruitment and DNA methylation regulation of the VSMC gene repertoire. Besides recruitment, methylation modifiers can also directly and indirectly influence the expression of other methyltransferases and demethylases. Chatterjee et al. demonstrated that methyltransferase SUV39H1 mediates repressive chromatin conformation at *KDM4A* [46]. The repressive SUV39H1-mediated enrichment in H3K9me2 on *KDM4A* leads to a decrease in KDM4A-mediated removal of H3K9 methyl groups on the VSMC contractile genes, their subsequent repression, and triggers VSMC transition to a dedifferentiated and proliferative phenotype. RNA methylation has been mainly associated with the regulation of transcript stability, splicing, and translation. Recent studies have, however, linked this mechanism to the regulation of gene activation of DNA methylation [173,174]. For example, METTL3 can recognize CpG motifs and mediates the recruitment of TET1 to promote DNA demethylation at these loci [154]. While these studies present interesting instances of crosstalk between methylation enzymes and highlight the complexity of methylation-mediated gene regulation, there remain notable gaps in our current understanding of VSMC epigenetic programming that accounts for coordinated regulation of the histone-DNA-RNA methylation axis.

## 7. Elucidating Non-Canonical Functions of Methylation Regulators

Recent studies have demonstrated that HMT, HDM, and TET function can be independent of their canonical catalytic activity in specific pathological, developmental, and regulatory contexts. Lysine-specific demethylase 1 (LSD1) is notably associated with non-canonical mechanisms that are not reliant on its demethylase activity. LSD1 was shown to activate genes in cancerous prostate tumors and promote prostate cancer cell survival [175]. LSD1 suppression in prostate cancer cells did not significantly alter H3K4me2 and H3K9me2 enrichment on LSD1-bound, differentially expressed genes. Furthermore, catalytically inactive LSD1 was sufficient to rescue expression of the cancer gene signature in LSD1-depleted cancer cells. LSD1 demethylase-independent function was also implicated in embryonic stem cells (ESC) cell fate transition [176]. LSD1 catalytic deficiency did not significantly contribute to gene dysregulation in ESC and was shown to have little impact on epiblast-like cell and embryoid body differentiation of ESC. LSD1 was further proposed to serve a demethylase-independent scaffolding function to sustain DNMT1 and UHRF1 protein stability in mouse ESC [177]. The histone methyltransferases DOT1L and SET1A have also been shown to employ non-canonical mechanisms during cell fate transitions. Catalytically inactive DOT1L was shown to have a limited effect on the neural differentiation of ESCs compared to DOT1L-depleted cells [178]. DOT1L catalytic inactivation did not significantly alter the expression of neural progenitor cell markers, including *Nestin*, *Sox11*, *Sox9*, and *Cd117*. Methyltransferase-dependent and independent activity of SET1A appears to be context-dependent in ESC [179]. SET1A catalytic activity was not required for ESC proliferation and self-renewal. However, ESCs expressing catalytically deficient SET1A were unable to undergo embryoid body differentiation. MLL3, MLL4, and JMJD2 were shown to have catalytic-independent functions in transcriptional regulation. Catalytically dead MLL3/4 ESC retained Polymerase II occupancy at MLL3/4-bound enhancers compared to MLL3/4-depleted ESC [180]. Recently, JMJD2 was shown to regulate enhancer-promoter interactions and the looposome independently of demethylase activity [181]. JMJD2 appears to regulate enhancer-promoter interactions through the formation of condensates at enhancer-promoter interaction sites, which JMJD2 maintains independently of H3K9me3, H3K9me2, and H3K36me3 modification. Finally, recent studies have shown that TET2 can regulate cellular functions through a non-canonical, dioxygenase-independent role [182,183]. Mechanistically, TET2 can recruit other epigenetic modifiers such as HDAC2 or O-Linked N-Acetylglucosamine (GlcNAc) Transferase to promote gene repression or activation, respectively [153,184]. The potential non-canonical and non-catalytic role of methylation-regulating enzymes in controlling VSMC function and atherosclerosis pathogenesis remains to be evaluated.

## 8. Conclusions and Remaining Questions

Evidence links multiple histone, DNA, and RNA methyltransferases/demethylases to regulatory mechanisms that direct VSMC phenotypic transition (including direct regulation of VSMC contractile genes), proliferation, plasticity, and participation in atherosclerosis. Further characterization of methylation status and methylation enzyme function in VSMC would benefit from re-examination of the methods applied in current studies. A lack of in vivo inducible SMC-specific KO and fate mapping models was apparent in the body of literature reviewed. Results of non-inducible VSMC KO models may be impacted by the consequences of methylation enzyme ablation in VSMC during development. In contrast, overexpression of methyltransferase/demethylase can bias results due to alterations in enzyme/substrate stoichiometry. Epidrugs have also been used to assess the effects of methylation enzyme inhibition in preclinical models of atherosclerosis. However, the broad inhibition of methylation enzymes with epidrugs does not circumvent the need to precisely delineate the contribution of SMC-specific methylation status and methylation enzyme function to atherosclerosis progression. Alongside inducible and SMC-specific KO models, temporal regulation of methyltransferase/demethylase expression should be considered during investigation. The histone, DNA, and RNA methylation landscapes are dynamic and finely tuned to cues that may occur in early or later stages of vessel injury. Clarifying the temporal relationship between methylation enzyme expression and methylation status in injury and disease states would reconcile conflicting findings on methylation enzyme expression. Contrasting findings across studies are not limited to differences in methyltransferase/demethylase expression. Methyltransferase and demethylase regulation of VSMC phenotypic transition and plasticity is highly context dependent. Methyltransferases/demethylases target different substrates and interact with distinct transcription factors, chromatin modifiers, and chaperones in a context-dependent manner. Of particular interest, methylation enzymes can also function independently of catalytic activity and participate in non-canonical mechanisms. In atherosclerosis, upstream regulatory mechanisms, including inflammation, metabolism, and hypoxia, may contribute to potential methyltransferase/demethylase non-canonical mechanisms that are linked to pathogenesis.

## Figures and Tables

**Table 1:** Methylation enzymes and readers involved in the regulation of contractile genes or proliferation/inflammation genes in VSMC and atherosclerosis pathogenesis. The table also reports studies investigating the relevance of these proteins in pharmacological studies employing epidrugs for their inhibition during vascular injury or disease. n/k: not known.

	Name	Gene	Methylation	Regulation of SMC Contractile Genes	Regulation of Proliferation/ Inflammation Genes	Atherosclerosis Studies	Epidrugs
Histone methyl-transferases	SMYD2	SMYD2	H3K4me1/3	Yes [38]	Yes [39]	-	-
	SMYD3	SMYD3	H3K4me1/3	n/k	Yes [43,44]	-	-
	DOT1L	DOT1L	H3K79me2	n/k	Yes [45]	[45]	-
	SUV39H1	SUV39H1	H3K9me3	Yes [46,47]	n/k	-	-
	G9A	EHMT2	H3K9me1/2	n/k	Yes [48]	-	UNC0638 [48]
	GLP	EHMT1	H3K9me1/2	n/k	Yes [48]	-	UNC0638 [48]
	EZH2	EZH2	H3K27	Yes [49,50]	n/k	[51,52]	GS126 [53], GSK343 [49], UNC1999 [54]
Histone demethylases	JMJD1A	KDM3A	H3K9me2	Yes [55]	n/k	-	-
	JMJD2A	KDM4A	H3K9me2	Yes [46]	n/k	-	-
	LSD1	KDM1a	H3K4me1/2	n/k	n/k	[56]	GSK2879552 [56]
			H3K9me				
DNA methyltransferases	DNMT1	DNMT1	5mC	Yes [57,58]	n/k	-	5-azacytidine [57]
	DNMT3A	DNMT3A	5mC	Yes [59]	n/k	-	5-azacytidine [57]
DNA methyl cytosine dioxygenase	TET2	TET2	5hmC	Yes [60]	n/k	-	-
	TET1	TET1	5hmc	n/k	Yes [61]	-	-
DNA methylation reader	UHRF1	UHRF1	5mC/5hmC	n/k	Yes [62]	[62]	-
RNA methyltransferases	METTL14	METTL14	m^6^A	n/k	Yes [63,64]	[65]	-
	METTL3	METTL3	m^6^A	n/k	Yes [66,67,68]	[69,70]	-
RNA Reader	YTHDF1	YTHDF1	m^6^A	n/k	n/k	-	-
	YTHDC2	YTHDC2	m^6^A	n/k	n/k	-	-

## Data Availability

No new data were created or analyzed in this study.
